# Loss of the neurodevelopmental disease-associated gene *miR-146a* impairs neural progenitor differentiation and causes learning and memory deficits

**DOI:** 10.1186/s13229-020-00328-3

**Published:** 2020-03-30

**Authors:** Julien Fregeac, Stéphanie Moriceau, Antoine Poli, Lam Son Nguyen, Franck Oury, Laurence Colleaux

**Affiliations:** 1 Developmental Brain Disorder Laboratory, INSERM UMR 1163, Imagine Institute, Paris, France; 2 Paris Descartes–Sorbonne Paris Cité University, Paris, France; 3 Institut Necker Enfants Malades (INEM), Institut National de la Santé et de la Recherche Médicale (INSERM) U1151, 75015 Paris, France

**Keywords:** Neurodevelopmental disorders, MicroRNA, miR-146a, Neurodevelopment, Neural stem cells differentiation, Hippocampus-dependent cognitive functions

## Abstract

**Background:**

Formation and maintenance of appropriate neural networks require tight regulation of neural stem cell proliferation, differentiation, and neurogenesis. microRNAs (miRNAs) play an important role in brain development and plasticity, and dysregulated miRNA profiles have been linked to neurodevelopmental disorders including autism, schizophrenia, or intellectual disability. Yet, the functional role of miRNAs in neural development and postnatal brain functions remains unclear.

**Methods:**

Using a combination of cell biology techniques as well as behavioral studies and brain imaging, we characterize mouse models with either constitutive inactivation or selectively hippocampal knockdown of the neurodevelopmental disease-associated gene Mir146a, the most commonly deregulated miRNA in developmental brain disorders (DBD).

**Results:**

We first show that during development, loss of *miR-146a* impairs the differentiation of radial glial cells, neurogenesis process, and neurite extension. In the mouse adult brain, loss of *miR-146a* correlates with an increased hippocampal asymmetry coupled with defects in spatial learning and memory performances. Moreover, selective hippocampal downregulation of *miR-146a* in adult mice causes severe hippocampal-dependent memory impairments indicating for the first time a role for this miRNA in postnatal brain functions.

**Conclusion:**

Our results show that *miR-146a* expression is critical for correct differentiation of neural stem cell during brain development and provide for the first time a strong argument for a postnatal role of *miR-146a* in regulating hippocampal-dependent memory. Furthermore, the demonstration that the *Mir146a*^*−/−*^ mouse recapitulates several aspects reported in DBD patients, including impaired neurogenesis, abnormal brain anatomy, and working and spatial memories deficits, provides convincing evidence that the dysregulation of *miR146a* contributes to the pathogenesis of DBDs.

## Background

MicroRNAs (miRNAs) are endogenous short single-stranded non-coding RNAs of ~20–22 nucleotides that play a key role in gene expression regulation by targeting specific messenger RNA (mRNA) for degradation or translation repression [1]. miRNA genes are first transcribed as primary transcripts that consist of one or more hairpin structures. This transcript is cleaved into short pre-miRNA transcript that contains a stem-loop and subsequently exported to the cytoplasm. Following export, pre-miRNA is processed by the endonuclease Dicer to generate a mature miRNA duplex and both strands derived from this duplex (-5p and -3p) can be loaded into the Argonaute proteins so as to generate the effector RISC complex. miRNAs function as master regulators as each of them interacts with hundreds of mRNA transcripts. miRNAs are expressed throughout embryonic development and life of most eukaryotic organisms and take part in almost every cellular and developmental process investigated so far.

miRNAs are particularly important for proper brain development as demonstrated by the dramatic apoptotic phenotypes of mouse mutants abolishing all miRNAs synthesis [2]. Fifty percent of all identified miRNAs are expressed in the mammalian brain [3] where they are differentially distributed in distinct regions and cell types. miRNAs regulate multiple aspects of brain development, including neural stem cell proliferation and differentiation, cell subtype determination, neurogenesis, and synapse formation [4]. Moreover, synapse stability and plasticity are regulated by local interactions between miRNAs and target mRNAs. Despite rapid progress in miRNA discovery, understanding the in vivo implication of single or subnetworks of miRNAs in specific cellular contexts, and the biological relevance of their predicted miRNA–mRNAs interactions remain challenging. The use of conventional genetic approaches is difficult, in particular in mammals, because of redundancy (multiple miRNA loci encoding the same mature form) and the small size of the loci, making them poor targets for mutation. Thereby, most insights into the role of miRNAs in neural development were obtained, in cell culture and animal models, by investigating the effect of miRNAs silencing by miRNA antagonist or the impact of miRNA overexpression by miR Mimics.

Over the past decade, dysregulated processing and altered abundance of a wide range of miRNAs have been associated with neurodegenerative disorders [5] and neuroblastoma [6]. Widespread changes in miRNA levels have also been observed in developmental brain disorders (DBDs) including autism spectrum disorders (ASD) [7], schizophrenia [8], intellectual disability (ID) [9], and epilepsy [10]. However, the precise miRNA-based mechanisms during brain development and in higher-order brain processing, including learning and memory and cognition, are still poorly understood.

Among the differentially expressed miRNAs identified by transcriptomic analysis in DBD patients, *miR-146a-5p* (referred to as *miR-146a* from now on) is one of the most commonly observed [7]. In ASD cases, it has been reported in five different cohorts and tissue types including post-mortem brain [11, 12] olfactory mucosal stem cells, fibroblasts [9], and lymphoblastoid cell line [13]. Its abnormal expression has also been reported in ID [9] and epilepsy [14]. *miR-146a* is 100% conserved between humans and mice; it is expressed broadly in the brain during embryonic development and restricted to neurons at postnatal stages. Importantly, modulating the expression of this particular miRNA was shown to reduce the latency, the duration as well as the intensity of the induced epilepsy in rodent models of telencephalon epilepsy [15, 16].

*miR-146*a is known for regulating NFκB [17], NOTCH [18], and WNT/β-catenin [19, 20] pathways in a tissue and developmental stage-dependent manner. Our group has also demonstrated the pro-neuronal effects of *miR-146a* overexpression in a H9 model of human neural stem cells [12]. Moreover, a body of in vitro works also suggests a role of *miR-146*a in neuron survival and apoptosis [21, 22], axonal growth [23], and AMPA receptor endocytosis [24]. Taken together, these data support the importance of *miR-146a* in cultured cells but give no indication either on its mechanism of action or on its physiological functions in vivo during neurodevelopment and at postnatal stages.

To investigate these aspects, we decided to study the impact of abnormal *miR-146a* expression level on mouse brain development and at adult stages. We focused on a mutant mouse line bearing a targeted deletion of the *miR-146a* gene [25] and characterized this *Mir146a*^*−/−*^ mouse model using a combination of imaging and cell biology techniques as well as behavioral studies. We first showed that loss of *miR-146a* leads to transient neurodevelopmental defects in the differentiation of apical radial glia (aRG) and neurogenesis. We next found that loss of *miR-146a* correlates with an increased hippocampal asymmetry in the adult brain coupled with defects in spatial learning and memory performances. Lastly, we showed that selective knockdown of *miR-146a* in the adult hippocampus leads to severe learning and memory impairments associated with a reduction of adult hippocampal neurogenesis, demonstrating for the first time that *miR-146a* play an important role in adult brain functions. Together, these results highlight the key roles of small non-coding RNA in the brain for the control of neuronal development and cognition and provide new insight into the mechanisms linking impaired *miR-146a* expression to DBDs.

## Methods

### Animal housing and ethics

For this study, we used the mouse line B6.Cg-Mir146^tm1.1Bal^/J (stock no. 016239) from Jackson Laboratory. Mice were housed and handled in accordance with current best practices. Their well-being was monitored daily by a contractor as a paid service (Animalliance). Homozygous mice at 6–8 months of age start to develop lymphoproliferative and myeloproliferative diseases, subsequently leading to anemia, thrombocytopenia, and lymphopenia ([25] and Jackson Laboratory). As such, all molecular and behavioral analyses described in this study were performed before mice reach 6 months old (4 months old at the latest for behavioral tests), before the appearance of any inflammatory disease signs. All experiments performed were approved by an independent ethics committee of Paris Descartes University (approval number 2015101215207380) and declared to the French Ministry of Research.

### ELISA analysis

Two ELISA kits were used to assess the concentration of Il-1β (15571957, Fischer Scientific) and TNF-α (15562017, Fischer Scientific). We tested the hippocampus and cortex of the first batch of mice used for the behavioral studies. Cells were lysed using the FastPrep system (MPbio) in EBC lysis buffer, followed by a 15-min centrifugation at 4 °C to remove debris. The supernatant containing the proteins were used for the tests that were performed following the manufacturer’s protocol.

### Brain magnetic resonance imaging

MRI were performed on a 4.7 Tesla MR scanner (Biospec 47/20 BrukerBiospin, Ettlingen, Germany) with a combination of a transmit volume coil and a surface receive branch coil. Anesthesia was induced with 4% isoflurane and maintained at 1% during imaging. The parameters of the spin-echo TurboRARE T2-weighted sequences are as follows: field of view (FOV) 2 × 1.5 cm; Matrix 200 × 150; resolution 100 × 100 μm; slice thickness 350 μm; 30 slices for the sagittal slices with TR/TE 5096/60 ms (11 min, 28 s); 46 slices for the axial slices with TR/TE 7850/60 ms (17 min, 40 s); RARE factor 9. All four mutant mice were males whereas the control group was constituted of one female and two males.

### BrdU injection and detection

E14.5 heterozygous *Mir146a*^*+/−*^ pregnant mice were injected with BrdU (Thermo Fisher) at 28 mg/kg and dissected 2 h after injection for pulse chase or at P7 for birth dating analysis. Brains were fixed in 4% PFA (Antigenfix, Diapath) during 24 h at 4 °C, washed 3 times in PBS and incubated 24h in PBS–30% sucrose. Brains were then frozen in O.C.T. compound (Tissue-Tek SAKURA) and stored at – 80 °C. Forteen micrometers sagittal sections were cut using a cryostat (Thermo Fisher) and mounted onto Fisher Superfrost Plus slides. Sections were incubated in 2N HCl for DNA denaturation at 37 °C for 30 min then washed 3 times with 0.1% Triton X-100 (Sigma) in PBS. Blocking was realized using 10% Normal Goat Serum (Clinisciences) and 0.1% Triton X-100 in PBS. Slides were incubated overnight at 4 °C with a rat anti-BrdU antibody (ab6326, Abcam). Slides were then washed 3 times with 0.1% Triton X-100 in PBS and incubated with Alexa fluor 488 donkey anti-rat antibody (A21208, Thermo Fisher) at room temperature for 2 h. Slides were finally washed three times in PBS, mounted using the Prolong gold antifade reagent with DAPI (P36935, Thermo Fisher) and scanned in a Nanozoomer 2.0 (Hamamatsu). 250μm × 250 μm images were taken in the neocortex and horizontally binned in 5 layers of equal size; for birth dating 1000 × 800 μm images were used. BrdU^+^ cells were counted in each bin or layer.

### PH3, PAX6, TBR2, and NeuN immunofluorescence and quantification

Pups were sacrificed at E14.5 and the embryonic brains were fixed, frozen, and cut into sagittal sections as previously described. An antigen retrieval step was performed by incubating the slides with 10mM Sodium Citrate buffer 10 min at 100 °C. Slides were then allowed to cool down and rinsed once with PBS before blocking 1h at room temperature with 10%NGS and 0.1% Triton X-100 in PBS. Anti-PH3 antibody (06-570, Merck Millipore) and anti-NeuN (ab177487, Abcam) were used alone while anti-PAX6 (901301, Biolegend) and anti-TBR2 (14-4875-80, ThermoFisher) antibodies were used concomitantly for co-staining. In all conditions, primary antibody was incubated overnight at 4 °C. After three washes with 0.1% Triton X-100 in PBS, slides were incubated with a matching secondary antibody (Alexa fluors, Thermo Fisher). Slides were then washed three times in PBS and mounted and scanned as previously described. We counted the PH3^+^ cells at the apical surface and in the SVZ independently in images of 650 μm of width and calculated the percentage of PH3^+^ cells at the SVZ. 250μm × 250 μm images were taken in the neocortex for the PAX6-TBR2 co-staining. Each cell type was counted independently. To analyze the NeuN labeling at E14.5, the thickness of the NeuN+ layer was measured at least four times along the neocortex.

### Doublecortin (DCX) immunohistochemistry

For DCX immunohistochemistry, brain sections (25 μm) were washed in 0.1 M PBS and then quenched in 0.3% H_2_O_2_ in 0.1 M PBS/CH_3_OH (1:1) for 15 min at room temperature. Sections were then washed and blocked in 10% normal donkey serum in 0.1 M PBS with 0.5% Triton X-100 for 2 h at room temperature. Incubation with primary antibody was performed at 4 °C overnight (Rabbit Anti-Doublecortin antibody (ab18723)) in 0.1 M PBS with 0.5% Triton X-100. Sections were then washed in 0.1 M PBS and incubated with a biotinylated secondary antibody (donkey anti-rabbit; 1:250, Jackson ImmunoResearch, West Grove, PA) for 2 h at room temperature. Sections were then washed in 0.1 M PBS and treated next with avidin-biotin-peroxidase complex (ABC Elite Kit, Vector Labs, Burlingame, CA) followed by a 3,3′diaminobenzidine as a substrate for staining (Vector, Burlingame, CA).

### Primary neuronal culture and neurite outgrowth measurement

Primary cortical neurons were extracted from E17.5 embryos using as previously described. Approximately 30,000 cells were plated per well in the 96-well plate for live cell imaging (IncuCyte® system, Essen Bioscience). For each well, 9 images were taken at fixed spots every 3 h during 6 days. Media was change 1 day after plating to remove debris, then every 3 days thereafter. In post analysis, a mask was designed using the inbuilt Neurotrack software module to detect neurites and cell body. The same mask was applied to all time points and all repeats to record neurite extension over time.

### Adeno-associated viruses expressing shRNA

Adeno-associated viruses (AAV) expressing shRNA were purchased from Applied Biological Materials Inc (Richmond, Canada). shRNAs specific to mir146a (mmu-miR-146a-5p AAV miRNA Virus, serotype 9), or scrambled non-targeting negative control (AAV-miR-Off-Blanck Control Virus, serotype 9) were injected in a volume of 1 μl (bilaterally), 3 weeks prior to behavioral tests. The AAV titers were > 1 × 10^9^GC/ml.

### Stereotaxic surgery

Mice were anesthetized by intraperitoneal injection of ketamine hydrochloride (20 mg/ml BW) (1000 Virbac) and xylazine (100mg/ml BW) (Rompun 2%; Bayer) and placed in a stereotaxic frame (900SL-KOPF). Ophthalmic eye ointment was applied to the cornea to prevent desiccation during surgery. The area around the incision was trimmed and Vétoquinol was applied. AAV shRNA were injected bilaterally into the dorsal hippocampi using the following coordinates from Bregma (Paxinos and Franklin, 2008): X = ± 1.4 mm, Y = 2.0 mm, and Z = − 1.33 mm. A 1-μl volume of AAV was injected stereotaxically over 4 min (injection rate 0.25 μl per min). To limit reflux along the injection track, the needle was maintained in situ for 4 min between each 1 μl injection.

### 3-foot shock contextual fear conditioning (CFC)

Mice were transported a short distance from the holding mouse facility to the testing room in their home cages and left undisturbed for at least 1 h before the beginning of the test. The conditioning chambers were obtained from Bioseb (France) with internal dimensions of 25 × 25 × 25 cm. Each chamber was located inside a larger, insulated plastic cabinet that provided protection from outside light and noise (67 × 55 × 50 cm, Bioseb, France), and mice were tested individually in the conditioning boxes. Floors of the chamber consisted of 27 stainless steel bars wired to a shock generator with scrambler for the delivery of foot shock. Signal generated by the mice movements was recorded and analyzed through a high sensitivity weight transducer system. The analog signal was transmitted to the Freezing software module through the load cell unit for recording purposes and analysis of time active/time immobile (Freezing) was performed. The CFC procedure took place over two consecutive days. On day 1, mice were placed in the conditioning chamber, and received 3 foot-shocks (1 s, 0.5 mA), which were administrated at 60, 120, and 180 s after the animals were placed in the chamber. They were returned to their home cages 60 s after the final shock. Contextual fear memory was assessed 24 h after conditioning by returning the mice to the conditioning chamber and measuring freezing behavior during a 4-min retention test. Freezing was scored and analyzed automatically using Packwin 2.0 software (Bioseb, France). Freezing behavior was considered to occur if the animals froze for a period of at least two seconds. Behavior was scored by the Freezing software and analyzed by two observers blind to mouse treatment (LSM and SM).

### Novel object recognition paradigm (NOR)

We used a modified version of the NOR task described by Ennaceur and Delacour in 1988 [26]. Mice were transported a short distance from the holding mouse facility to the testing room in their home cages and left undisturbed for at least 1 h before the beginning of the test. The testing room was lit with two 60 W light bulbs and behavior sessions were recorded with a camera above the testing arena (grey plastic box (60 × 40 × 32 cm)). Mice could not contact or see each other during the exposures. The light intensity was equal in all parts of the arena (approximately 20 lx). Two different objects were used, available in triplicate. The objects were (1) a blue ceramic pot (diameter 6.5 cm, maximal height 7.5 cm) and (2) a clear, plastic funnel (diameter 8.5 cm, maximal height 8.5 cm). The objects that serve as a novel object, as well as the left/right localization of the objects, were counterbalanced within each group. The objects elicited equal levels of exploration as determined in pilot experiments and training phase. Between exposures, mice were held individually in standard cages, the objects and arenas were cleaned with phagosphore, and the bedding replaced.

The NOR paradigm consists of three phases (over 3 days): a habituation phase, a training phase, and a testing phase. Mice were always placed in the center of the arena at the start of each exposure. On day 1, the habituation phase, mice were given 5 min to explore the arena, without any objects and were then taken back to their home cage. On day 2, the training phase, mice were allowed to explore, for 10 min, two identical objects arranged in a symmetric opposite position from the center of the arena and were then transported to their home cage. On day 3, the testing phase, mice were given 15 min to explore two objects: a familiar object and a novel one, in the same arena, keeping the same object localization.

The following behaviors were considered as exploration of the objects: sniffing, licking, or touching the object with the nose or with the front legs or directing the nose to the object at a distance ≤ 1 cm. Investigation was not scored if the mouse was on top of the object or completely immobile. The preference index for the novel object was calculated as (time spent exploring the new object/the total time spent exploring both objects), and the discrimination index was calculated as (time spent exploring the new object − time spent exploring the familiar object)/(total time spent exploring both objects). As a control, preference index for the (right/left) object location or for the object A versus B during the training phase of the NOR was measured in all groups of mice exposed to the test. We confirm here that no initial preference for any exposed object (A or B) or any orientation (right/left) was observed in any groups. The locomotion was assessed for each mouse. Behavior was scored on videos by two observers blind to treatment and the total exploration time of the objects was quantified in the training and testing phases (AP and SM).

### Object location memory test (OLM)

For the object location memory task, all procedures were identical to the novel object recognition task except that during the testing phase, rather than presenting a novel object, mice encountered both familiar objects, with one object located in a different place in the arena. The time and frequency of exploration of the novel/relocated object is measured as an index of memory. As a control, preference index for the (right/left) object location during the training phase of the OLM was measured in all groups of mice. We confirm here that no initial preference for any orientation (right/left) was observed in any groups. Behavior was scored on videos by two observers blind to treatment and the total exploration time of the objects was quantified in the training and testing phases (LSM and SM).

### Light-to-dark transition test (D/LT)

This test is based on the innate aversion of rodents to brightly illuminated areas and on their spontaneous exploratory behavior in response to the stressor that light represents. The test apparatus consists of a dark, safe compartment and an illuminated, aversive one. The lit compartment was brightly illuminated with an 8 W fluorescent tube (1000 lx). Naive mice were placed individually in the testing chamber in the middle of the dark area facing away from the doorway to the light compartment. Mice were tested for 10 min, and two parameters were recorded: time spent in the lit compartment and the number of transitions between compartments, indices of anxiety-related behavior and exploratory activity. Behavior was scored using an infrared light beam activity monitor using actiMot2 Software (PhenoMaster Software, TSE), and it was statistically analyzed using Prism program.

### Open-field est (OFT)

This test takes advantage of the aversion of rodents to brightly lit areas. Each mouse is placed in the center of the OFT chamber (43 × 43 cm chamber) and allowed to explore for 30 min. Mice were monitored throughout each test session by infrared light beam activity monitor using actiMot2 Software (PhenoMaster Software, TSE). The overall motor activity was quantified as the total distance traveled (ambulation). Anxiety was quantified by measuring the time and distance spent in the centerversus periphery of the open-field chamber.

## Results

### Altered balance between aRG and IP populations in the Mir146a^−/−^ neocortex

**Fig. 1:**
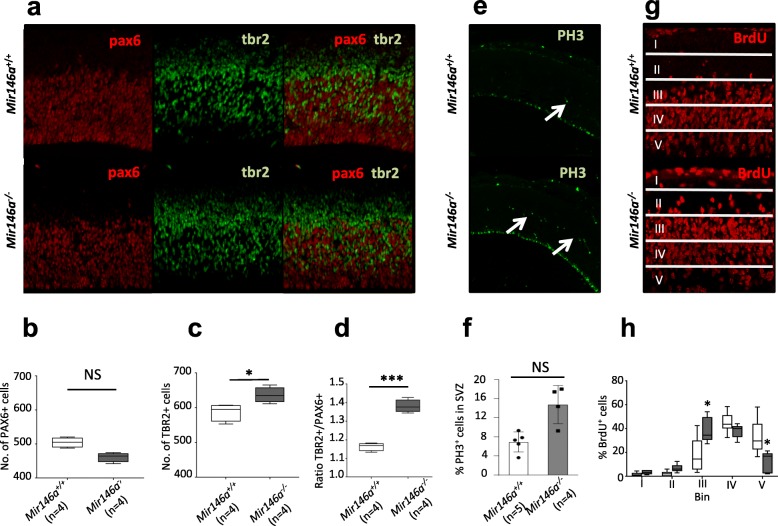
Altered aRG and IP population size in the *Mir146*^*−/−*^ brains. **a** Sagittal sections from *Mir146a*^*+/+*^and *Mir146a*^*−/−*^ brains at E14.5 labeled with PAX6, TBR2, and merge. **b** Boxplot showing the number of PAX6^+^ cells (±S.D.) counted using 3 images from comparable plane per mouse. **c** Boxplot showing the quantification of TBR2^+^ cells (±S.D.) from the same images as **b**. **d** Boxplot showing the ratio of TBR2^+^**/**PAX6^+^ cells. **e** Phospho-histone 3 (PH3) stains dividing cells in the neocortex at E14.5 (white arrows). **f** Graph showing the average number of PH3^+^ cells (±S.D.) in the SVZ. *P < 0.05 by Student’s unpaired 2-tailed *t* test. **g** BrdU staining in the neocortex at E14.5 after 2h pulse chase; images were divided into five equal bins for counting. **h** Percentage of BrdU+ cells (±S.D.) per bin (normalized against the total number of labeled cells). NS not significant; *p ≤ 0.05, **p ≤ 0.01, ***p ≤ 0.001 by Mann-Whitney *U* test

*miR-146a* has been shown to regulate the balance of cell cycle progression and differentiation of cultured progenitor cells in vitro [12]. Yet, we observed that the number of cortical neurons is unaltered in the adult *Mir146a*^*−/−*^ brain (Figure S1, Additional file 1), suggesting that, in vivo, there is no major defect in proliferation of neural progenitors. To resolve this discrepancy, we quantified the ratio between the two main neuronal progenitor cell types that coexist in the mouse necortex at E14.5: apical radial glia (aRG) and intermediate progenitors (IP) (Figure S2, Additional file 1). We performed immuno-stainings of the neocortical slices for PAX6 and TBR2 in order to label aRG and IP, respectively (Fig. 1a). *Mir146a*^−/−^ embryos showed an imbalance of aRG/IP cell populations as they exhibit a 10% decrease of PAX6^+^ cells (Fig. 1b), and a 10% increase of TBR2^+^ cells (Fig. 1c) compared to control littermates. Consequently, the difference in ratio of TBR2^+^/PAX6^+^ cells was significantly (*p* = 0.00012) altered in mutant mice in comparison to controls (Fig. 1d).

aRG and IP undergo mitosis at distinct locations as RG cells divide in the ventricular zone (VZ) to produce IP cells, while IP cells divide in the subventricular zone (SVZ) to produce pairs of daughter neurons. We thus stained the neocortex for the mitosis marker Phospho-Histone 3 (PH3) (Fig. 1e). In the mutant neocortex, there was no difference in the number of PH3^+^ cells at the apical surface (where aRG divide) (Figure S3, Additional file 1). However, we observed a significant increase of PH3^+^ cells in the SVZ (where IP divide) (Figure 1f). To further corroborate these findings, we assessed the position of the cells that are progressing through S phase by BrdU pulse chase. We counted their distribution in the neocortex in five equal bins and observed a shift towards the basal lamina in the mutants compared to WTs (Fig. 1g). Quantification of BrdU^+^ cells in each bin showed a significant increase in bin III and a significant decrease in bin V (Fig. 1h), corresponding to the SVZ and the apical surface, respectively. Collectively, these results suggest an altered balance between the self-renewal of radial glial stem cells and the production of their descendent progenitor cells in *Mir146a*^−/−^ neocortex.

### Loss of *miR-146a* causes defects in neuronal differentiation and neurite outgrowth

**Fig. 2:**
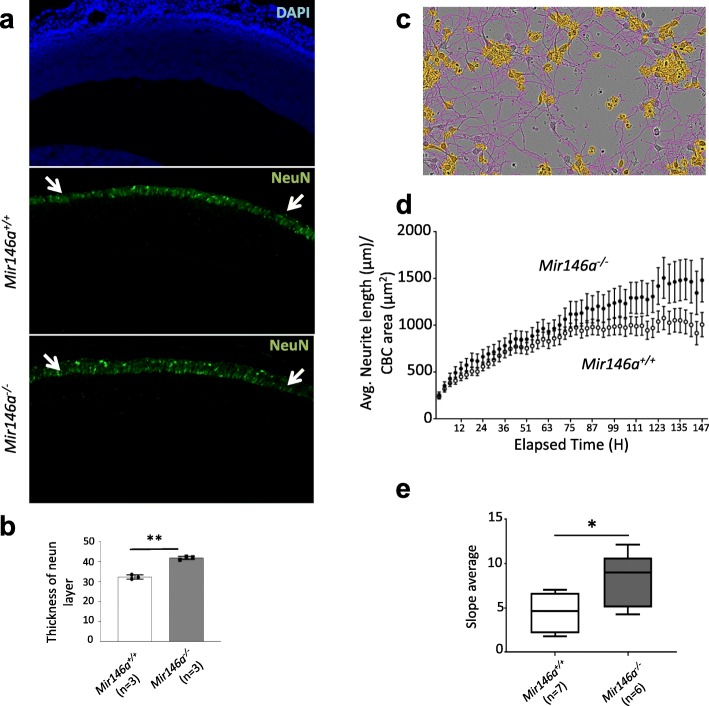
Altered neurogenesis in the *Mir146a*^*−/−*^ brains. **a** Sagittal section of E14.5 neocortex of *Mir146a*^*+/+*^ and *Mir146a*^*−/−*^ brain stained with DAPI and the post-mitotic neuronal marker NeuN; white arrows show the differentiated neuron layer where the measurements were made. **b** Graph comparing the average thickness (±S.D.) of the NeuN^+^ cells layer between WT and mutant embryos; at least 3 images from comparable plane per mouse were used for the analysis; **p < 0.01 by Student’s unpaired 2-tailed *t* test. **c** Automated phase contrast image segmentation of neurites (pink) and cell body clusters (CBC in blue) of *Mir146a*^*−/−*^ neurons at day 3 using the Neurotrack software module. **d** Graph shows the total length of neurites (±S.E.M) of neurons from *Mir146a*^*+/+*^ and (*n* = 7) *Mir146a*^*−/−*^ embryos (*n* = 6). Images were taken by the Incucyte S3 at 9 different spots per well (replicate) every 3 h during 6 days of culture; post analysis was performed automatically using the pre-designed mask; there are at least 4 technical replicates per embryo. **e** Graph shows the average of slopes (±S.D.) of neurite extension of corresponding graph **d**. **p < 0.01 by Wilcoxon ranked sum test

As neurogenesis relies on fine coordination between proliferation and differentiation of aRG, we tested whether the loss of *miR-146a* could impact the number and/or properties of newborn neurons in the neocortex. We, thus, stained the neocortical slices at E14.5 for the neuronal post-mitotic factor NeuN and measured the thickness of the neuronal layer (Fig. 2a). Consistent with previous results, a significantly enlarged NeuN^+^ cells layer (30% increase) was observed in the mutant neocortex compared to controls (Fig. 2b). As previously mentioned, this difference does not persist throughout brain development, as in the cortex of P30 and P60 of mutants and control *Mir146a*^*+/+*^ littermates, we did not detect any difference between the number of NeuN^+^ cells (Figure S1, Additional file 1). To investigate whether the absence of *miR-146a* may alter the neurite outgrowth, we used live cell imaging to monitor neurite extension in cultured primary neuron for 1 week (Fig. 2c and Additional file 2: Tables S1). *Mir146a*^−/−^ neurons had an average neurite length that was constantly longer than the length of the neurites of the control neurons (Fig. 2d, e).

Neuronal differentiation and migration are two closely related processes essential for the formation of the multilayered cortical structure. Therefore, we assessed whether this defect in neurogenesis interferes with the migrating trajectory of neurons by BrdU birth dating technique. We labeled with BrdU newly born neurons at E14.5, which are destined for layer IV of the cortex [27], and studied their distribution in each cortical layer at P7. This analysis did not detect any obvious difference in the distribution of BrdU^+^ cells in any cortical layer between mutants and controls (Figure S4, Additional file 1). Taken together, these results suggest a pro-neurogenic impact of the loss of *miR146a* in the *Mir146a*^*−/−*^ neocortex, which do not lead to obvious malformation of the cortical structure.

### Impaired hippocampal anatomy in the *Mir146a*^*−/−*^ mice

**Fig. 3:**
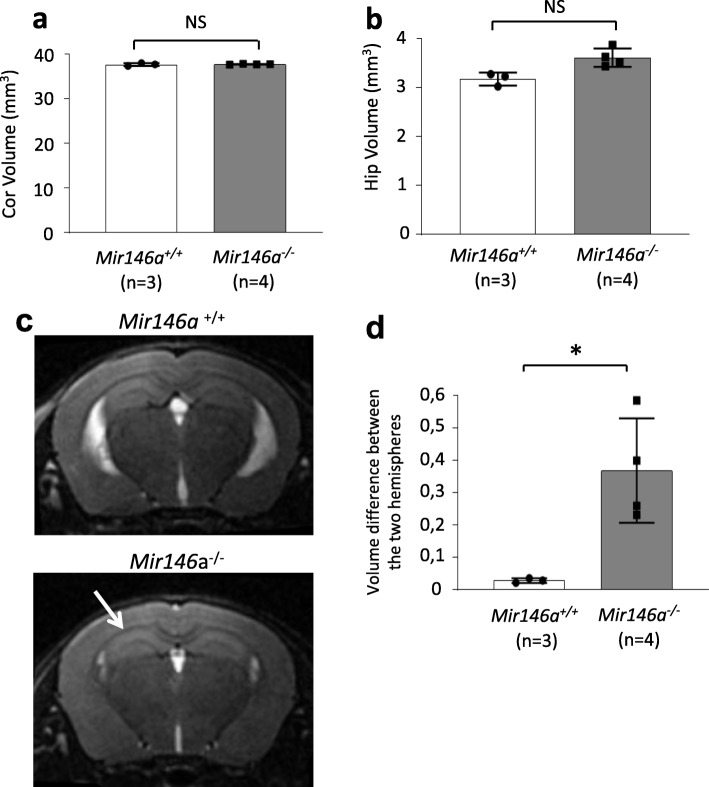
Increased hippocampus asymmetry in the *Mir146a*^*−/−*^ brain at P30. Volume (±S.D.) of the hippocampus (**a**) of the mutant and WT mice as measured by brain MRI at P30. The surface area of each region was measured from MRI images at 100 μM apart (5 images for the hippocampus and 15 for the cortex) and reconstructed using Image J to obtain final volume. **b** Brain MRI showing coronal sections. White arrows point towards the hippocampi; the larger white arrow point towards the enlarged hippocampus in the mutant. **c** Graph showing the volumes difference between two hippocampal hemispheres in *Mir146a*^*+/+*^ and *Mir146a*^*−/−*^ brains. *p < 0.05 by Student’s unpaired 2-tailed *t* test. On average, one hippocampus side is 15% larger than the other in *Mir146a*^*−/−*^ brains with random distribution (2 right > left and 2 left > right)

A wide spectrum of structural brain anomalies is frequently observed in DBD patients [28–30]. This includes white matter and/or temporal lobe abnormalities in children with non-syndromic ASD, mega cisterna magna in low-functioning autistic patients, brain hemispheric asymmetry in children with high-functioning autism and developmental language disorder, corpus callosum defects, cortical and/or cerebellar atrophy, and hippocampal alteration in intellectually delayed children. Structural brain anomalies were also described in mouse models of DBDs. Indeed, reduced volume of cerebellum as well as reduced thickness of corpus callosum were observed in *Mecp2-*null mice [31], while the Fragile X syndrome mouse model exhibits an increased volume of the parieto-temporal lobe and a smaller striatum [32]. To investigate whether such anatomical defects are present in the *Mir146a*^*−/−*^ mice, we performed magnetic resonance imaging (MRI) studies at P30, the earliest stage that allows us to measure the volume of the cortex and the hippocampus. While no significant difference was observed in the overall volume of these two regions (Fig. 3a, b) between mutant and control littermates, we observed a high hippocampal asymmetry between the two hemispheres in the mutant brains (Fig. 3c). In the *Mir146a*^*−/−*^ brains, one side is 15% bigger than the other with a random distribution regarding the enlarged side (2 mice with right > left and 2 mice with left > right) (Fig. 3d). The same analysis was also performed at P60 and similar results were observed (Figure S5, Additional file 1).

### Loss of *miR-146a* results in learning and memory deficits

**Fig. 4:**
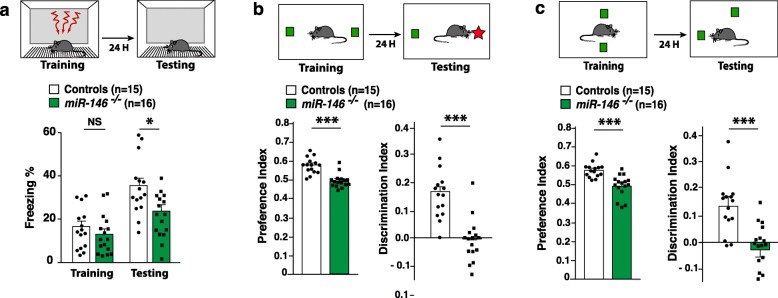
Behavioral analyses of the *Mir146a*^*−/−*^ mice*.***a** 3-foot shock CFC performed in a 3-month-old *Mir146a*^*−/−*^ mice (*n* = 16) and their control littermates (*n* = 15). Percent freezing was measured in CFC for each group during the training (as a control for the basal level of mouse freezing) and testing phases (to assess memory performances). **b** Novel object recognition test (NOR) performed in a 3-month-old *Mir146a*^*−/−*^ mice (*n* = 16) and their control littermates (*n* = 15). Discrimination and preference indexes were measured for each group during the testing phase. **c** Object location memory (OLM) task performed in a 3-month-old *Mir146a*^*−/−*^ mice (*n* = 16) and their control littermates (*n* = 15). Discrimination and preference indexes were measured for each group during the testing phase

In human, abnormal expression of *miR-146a* is observed in patients with cognitive deficits. Our MRI analyses performed in the *Mir146a*^*−/−*^ mice demonstrated a severe asymmetry of the hippocampus, a region of the brain playing a critical role in cognitive processes, such as learning and memory. Therefore, we subjected 3-month-old *Mir146a*^*−/−*^ mice and their controls littermates to a series of behavioral tests, that is thought to reflect behavioral functions related to hippocampus, measuring associative (one-trial contextual fear conditioning, CFC), episodic (novel object recognition test, NOR), and spatial (Object localization memory OLM) learning and memory (Fig. 4). In CFC, mutant mice exhibited no differences in baseline freezing time. However, ablation of *miR-146a* resulted in decreased context-elicited freezing time during the testing phase compared to their WT littermates, indicating that contextual fear memory is impaired in the mutant mice (Fig. 4a). Next, we used a modified version of the NOR paradigm [26, 33] that measures the rodent’s ability to recognize a novel object in the environment. WT mice are capable of differentiating novel objects from familiar ones and tend to explore novel ones for longer time. As shown in (Fig. 4b), 3-month-old *Mir146a*^*−/−*^ mice explored significantly less the novel object than WT controls. The same impairments were observed when memory was analyzed through the OLM task (assessing spatial learning and memory in rodents). Indeed, mutant mice explored significantly less the relocated object than WT littermates during the testing phase (Fig. 4c). Of note, mutant and WT mice had comparable performance in the open-field test (OFT) and light/dark paradigm (L/DT) (Figure S6, Additional file 1), indicating that their locomotion and anxious state were intact. Neuroinflammation is a well-recognized etiological component in ASD [34]. Given the role of *miR-146a* in innate and adaptive immune responses, we asked whether the behavioral anomalies observed in mutant mice could be related to altered neuro-immunoregulation. To that end, we tested by ELISA assays the level of inflammatory markers IL1-β and TNF-α in hippocampal and cortical brain lysates from the mice after the behavioral tasks. We did not detect any difference between the mutants and WT, excluding the confounding effects of inflammation in the observed behavioral defects (Figure S7, Additional file 1). Taken together, these data demonstrate that *miR-146a* constitutive inactivation significantly influences hippocampal-dependent spatial learning and memory performances, episodic memory and associative fear memory acquisition through a mechanism independent of the role of this miRNA in the regulation of inflammatory response.

### Postnatal knockdown of *miR-146a* affect adult neurogenesis and causes behavioral deficits

**Fig. 5:**
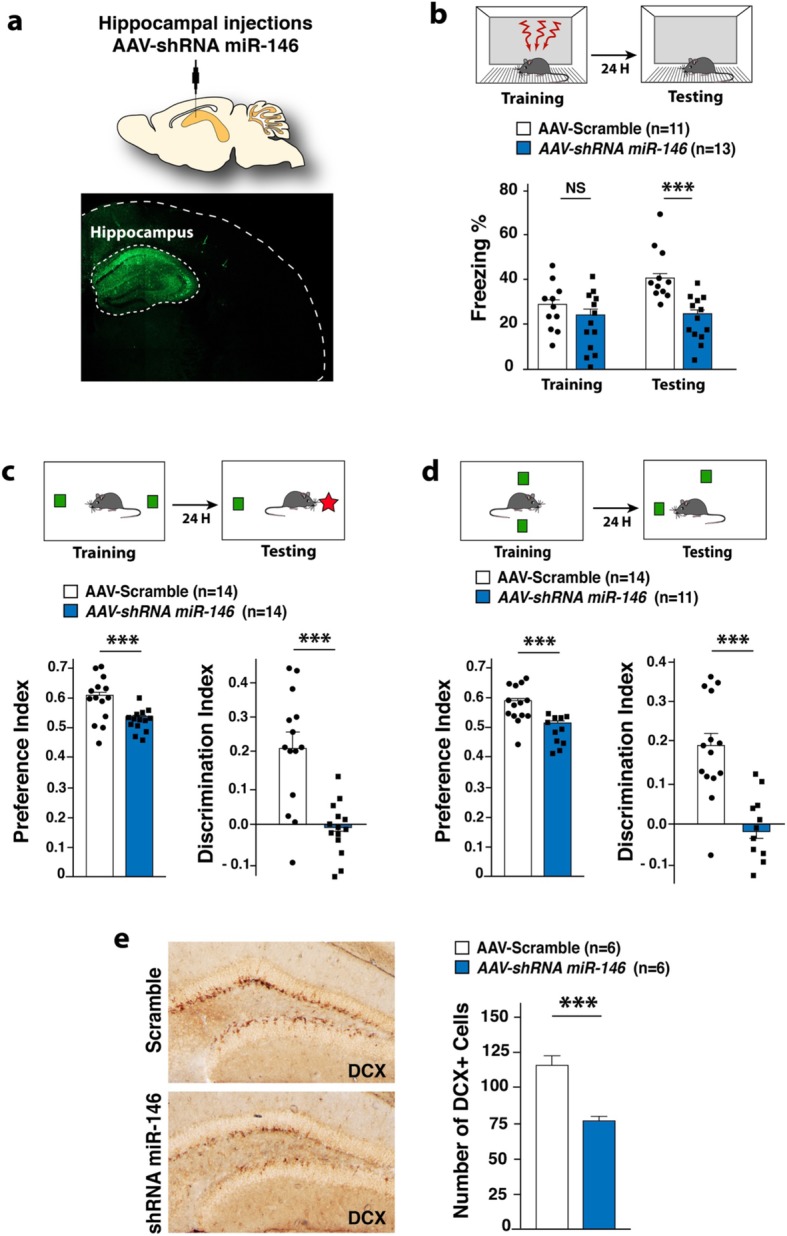
Behavioral analyses after selective hippocampal downregulation of *Mir146.***a** Fluorescent microscopy image of a coronal brain section collected 3 weeks after local stereotactic injections with AAV expressing shRNAmir for *Mir146a gene*. The construct expresses eGFP, which allow us to verify the site and the efficacy of infection. A Panoramic photo of an entire brain region was obtained by automatically aligning and stitching 50 tiled images into a mosaic (using the Zen Light Zeiss LSM software package). **b**–**d** Behavioral analyses (CFC (E), NOR (F), OLM (G)) performed in 3-month-old mice, 3 weeks after hippocampal stereotactic injections with either: AAV-*Mir146a*-shRNA or AAV-Scramble-shRNA. **e** Microtubule-associated protein doublecortin (DCX) immunohistochemistry showing a significantly lower number of DCX^+^ cells (mean number of DCX^+^ cells in the dentate gyrus (DG)/brain slides) in the DG of the mice locally injected with AAV-shRNA *miR 146a* in comparison to the one injected with AAV-shRNA scramble. All behavioral tests were performed on at least two independent experiments for each experiment: n6 mice per group. Results are given as mean ± s.e.m. *p ≤ 0.05, **p ≤ 0.01, ***p ≤ 0.001, NS: not significant; by Student’s *t* test compared to 3-month-old mice littermates. All behavioral tests were performed on at least two independent experiments for each experiment: n6 mice per group. Results are given as mean ± s.e.m. *p ≤ 0.05, **p ≤ 0.01, ***p ≤ 0.001, NS: not significant; by Student’s *t* test compared to 3-month-old mice littermates

Adult neurogenesis is known to play an important role in the maintenance of hippocampal memory capacity. We thus assessed the impact of postnatal mir-*146a-*knockdown on cognitive functions. To modulate *miR-146a* expression, we performed stereotactic injections of adeno-associated viruses (AAV) expressing shRNAs directed against *mir-146a* in the hippocampus of 2-months-old WT mice (Fig. 5a) and subjected mice to behavioral analyses 3 weeks after injections. As a result, we observed behavioral deficits in NOR, CFC, and OLM tests similar to those seen in *Mir146a*^*−/−*^ mice (Fig. 5b–d). Last, to get insight into the mechanisms underlying the observed behavioral phenotypes, we investigated the impact of *miR-146a* knockdown on hippocampal adult neurogenesis. The microtubule-associated protein doublecortin (DCX) is transiently expressed in proliferating progenitor cells and newly generated neuroblasts and is a reliable marker of adult neurogenesis level. As shown in Fig. 5e, we detected a drastic decrease of the number of DCX+ cells in the dentate gyrus of the mice locally injected with AAV-shRNA *miR-146*, demonstrating a reduced number of immature neurons in this region. Taken together, these findings demonstrate for the first time that the role of *mir-146a* in the brain is not restricted to neurodevelopmental processes, but that it also plays an important postnatal role in hippocampal neurons.

## Discussion

While many in vitro studies have been reported, the physiological role of miRNAs in brain development, cognitive functions and diseases has remained largely unexplored. Elucidating the specific cells and gene network regulated by each brain-expressed microRNAs is crucial for our understanding of both the mechanisms of neural circuit development and the etiology of neurodevelopmental disorders. A recent report demonstrated that in mouse partial loss of *miR-137*, a locus linked to autism and intellectual disability, impairs postnatal development and regulates synaptic plasticity by targeting the cyclic nucleotide phosphodiesterase family member Pde10a [35]. Here, we used mouse models with either constitutive inactivation or selectively hippocampal knockdown of the neurodevelopmental disease-associated gene *Mir146a* to evaluate the consequences of this inactivation on the differentiation and migration of neural stem cells, as well as on brain anatomy and cognition.

**Fig. 6:**
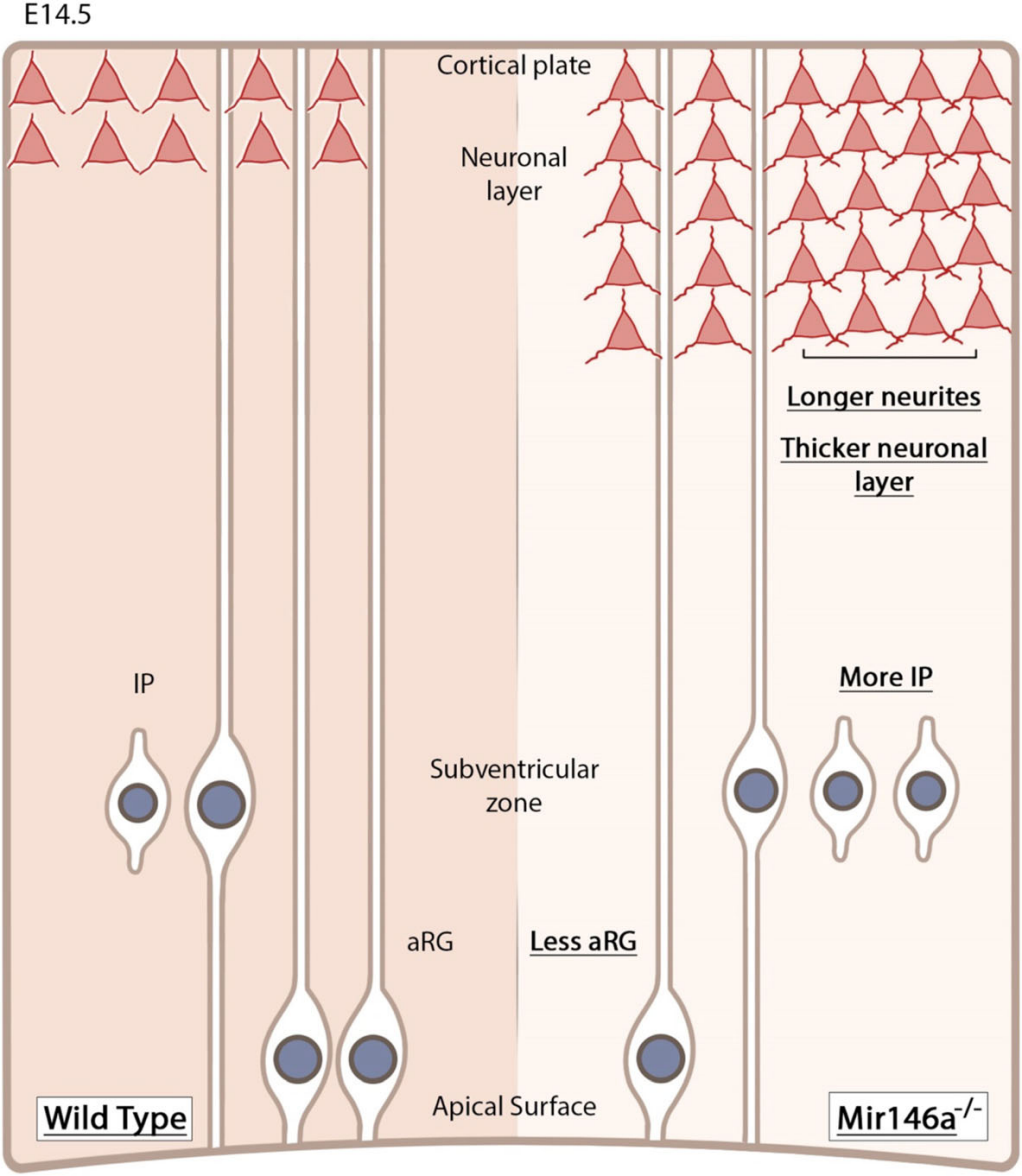
*miR-146a* promotes neuronal differentiation at E14.5. During neurogenesis, loss of *miR-146a* leads to imbalance of radial glial cells (aRG) and intermediate progenitor (IP) and enhances neuronal differentiation and neurite branching

We first show that proper *miR-146a* expression is critical for correct differentiation of neural stem cell during brain development (Fig. 6). These results are in line with the previously published in vitro studies demonstrating that increased *miR-146a* level enhanced neuronal differentiation in mouse NSC [36] and in H9 human neural stem cells [12]. Yet, while *miR-146a* overexpression in cultured NSC halts cell cycle progression and promotes neuronal differentiation through NOTCH pathway, how loss of *miR-146a* causes defective neuronal differentiation and which *miR-146a* target(s) may mediate these effects in the mutant brains is still an open question. In this regard, the adaptor protein NUMB protein is an excellent candidate. NUMB plays a key role in asymmetric division and cell fate determination in many cell types, including neural progenitors [37, 38]. NUMB is a validated direct target of *miR-146a* [39], and *miR-146a* was reported to facilitate activation of SHH signaling by targeting NUMB expression [40]. Various *Numb* transcript isoforms have been characterized that differ by the inclusion or not of exon3 and exon9. These isoforms display distinct developmental expression pattern and functions in the neuronal lineage [41]. The expression of the long NUMB isoform starts at E7, peaks at E10, and becomes undetectable by E13. By contrast, short isoform is present throughout development and in the adult brain. Moreover, while the long isoform promotes proliferation without affecting neuronal differentiation, the short one inhibits proliferation of the stem cells and promotes neuronal differentiation. Testing for NUMB transcript expression level in E14.5 neocortex and NUMB protein level by Western blot of aRG protein extracts did not reveal a significant difference between *Mir146a*^+/+^ and *Mir146a*^−/−^ samples. However, given the complexity of NUMB's function in brain development, and the role of NUMB isoform switch for neuronal development, we propose that loss of *miR-146a* in earlier stages of brain development may affect either short isoform NUMB protein level or NUMB isoform switch.

Besides the neurogenesis defects observed in early stages of brain development, no noticeable difference was observed in the number of neurons, the size, and the structure of the cortex between control and mutant brains at postnatal stages. This result suggests the establishment of compensatory mechanisms that prevent the production of supernumerary neurons and major structural abnormalities. However, we observed that *Mir146a*^−/−^ mice display an abnormal hippocampus structure with an increased hemispheric asymmetry that was not associated with any particular brain hemisphere. In human, the hippocampal volume is slightly larger in the right hemisphere both in children and in adults [42, 43]. While this asymmetry is sometimes lost in pathologies such as Williams syndrome [44] or Alzheimer’s disease [45], to our knowledge, an increased asymmetry with randomized distribution between right and left hemispheres was never reported. Although the mechanism underlying this defect remains unknown, especially its link with impaired radial glia differentiation and neurogenesis in the neocortex, this finding suggests that loss of *miR-146a* causes subtle alterations in brain anatomy. Perhaps the most intriguing finding is that *Mir146a*^−/−^ mice exhibited severe impairments in learning and memory functions without any locomotion or anxiety-related behavioral phenotype. Mechanistically, the deficit in learning and memory performances can be explained, at least in part, by the brain anatomical and neuronal differentiation defects observed in the mutant mice. However, our observation that postnatal downregulation of *mir-146a* specifically in the hippocampus region is sufficient to impair learning and memory capacities and causes altered neurogenesis indicates for the first time that this miR also have a postnatal role and is involved in hippocampus-dependent cognitive functions.

## Conclusion

Collectively, our data demonstrate that *miR-146a* inactivation alters processes involved in neural stem cell differentiation and provide for the first time a strong argument for a postnatal role of *miR-146a* in regulating hippocampal-dependent memory. The demonstration that the *Mir146a*^*−/−*^ mouse recapitulates several aspects reported in DBD patients, including impaired neurogenesis, abnormal brain anatomy, and working and spatial memories deficits, provides convincing evidence that impaired expression of *miR-146a* contributes to the pathogenesis of DBDs. Last, the relevance of the *miR146a*^-/-^ mouse model to study DBD opens the way for future investigation in the role of this miRNA in other key aspects of neurodevelopment such as glial cell biology, cognitive disorders and adult neurogenesis.

## Supplementary information

**Additional file 1: Figure S1.** The number of cortical neurons is unaltered in the adult *Mir146a*^*-/-*^ brain. (a) Cortical slides of WT (top panel) and *Mir146a*^*-/-*^ mouse (bottom panel) stained for NeuN to label post-mitotic neurons at P60. Number of NeuN^+^ cells normalized to area counted (±S.D.) in layer 2 (b), layers 3-4 (c), layer 6 (d) and all layers (e) in the WT and *Mir146a*^*-/-*^ cortical slices. At least 2 images at comparable plane per mouse were analyzed. The number of NeuN^+^ cells at P30 and P60 are indistinguishable, thus, we combined the results for each genotype. **Figure S2.** Neurogenesis in the mouse neocortex. Neuroepithelial cells (NE) proliferate up to E11 before differentiating into apical radial glia (aRG). aRG are located in the ventricular zone (VZ) where their nucleus undergo interkinetic nuclear migration (INM) and divide at the apical surface. aRG either self-replicate or give rise to a neuron or an intermediate progenitor (IP) after an asymmetric division. IP remain in the subventricular zone (SVZ) and can complete one or two cell cycles before differentiating into neurons. Neurons coming from aRG or IP migrate towards the intermediate zone (IZ) and the cortical plate (CP) where they acquire their final position and identity and start expressing post-mitotic markers such as NeuN. **Figure S3.** Number of PH3^+^ cells at apical surface of the E14.5 neocortex is unaltered in the *Mir146a*^*-/-*^. Graph showing the number of PH3^+^ cells (±S.D.) in images shown in Fig. 1e. NS, not significant by Student’s 2-tailed T-Test. **Figure S4.** Loss of *miR-146a* does not affect cortical layers organization. (a) BrdU signal at P7 labeled neurons born at E14.5. The white box shows the analyzed area. (b) Zoom of the white box from (a). The 6 layers of the mature cortex were determined and BrdU^+^ cells were counted using 3 images from comparable plane. (c) Graph shows the percentage of cells in each layer for the two genotypes, normalized against the total number of labeled cells. **Figure S5.** Increased hippocampus asymmetry in the *Mir146a*^*-/-*^ mice at P60. Volume (±S.D.) of the cortex (a) and the hippocampus (b) of the mutant and WT mice as measured by brain MRI at P60. (c) Scatterplot comparing the ratio between volumes of two hippocampal hemispheres in mutant and WT mice. NS: not significant Student’s unpaired 2-tailed T-Test. On average, one hippocampus side is 15% larger than the other in mutant brains with random distribution (2 right>left and 2 left>right). **Figure S6.** Locomotion and anxious state of the *Mir146a*^*-/-*^ mice are not affected. (a) Open field test (OFT) performed in 3-month old *Mir146a*^*-/-*^ mice (n=16) and their control littermates (n=15). Total distance (meter), % of the distance traveled in the center versus periphery, and % of the time spent in the center versus periphery were measured. (b) Light/Dark transition Test (L/DT) performed in 3-month old *Mir146a*^*-/-*^ mice (n=16) and their control littermates (n=15). The number of entries in the lit compartment, and % of the distance traveled and time spent in the lit compartment were measured. All behavioral tests were performed on at least two independent experiments, for each experiment: n≥6 mice per group. Results are given as mean ± s.e.m. *P ≤ 0.05, **P≤ 0.01, ***P ≤ 0.001, NS: not significant; by Student’s *t*-test compared to 3-month-old mice littermates. **Figure S7.** The *Mir146a*^*-/-*^ brain shows no sign of inflammation. Expression level (±S.D.) of inflammatory markers IL1-β (a and b) and TNF- α (c and d) in brain lysates of 8 WT and 8 mutant mice that were used for behavior studies. Technical quadruplicates were performed.

**Additional file 2: Tables S1.**

## Abbreviations

DBD: Developmental brain disorders
ASD: Autism spectrum disorders
ID: Intellectual disability
hNSC: Human neural stem cell
miRNA: MicroRNA
INM: Interkinetic nuclear migration
PH3: Phospho-histone 3
PAX6: *Paired box* 6 protein
TBR2: T-box brain protein 2
BrdU: Bromo-desoxy-uridine
DEG: Differentially expressed gene
CNS: Central nervous system
aRG: Radial glial cell
AAV: Adeno-associated viruses
IP: Intermediate progenitor
VZ: Ventricular zone
SVZ: Subventricular zone
WT: Wild type
