# Supplementary material for: Loss of the neurodevelopmental disease-associated gene miR-146a impairs neural progenitor differentiation and causes learning and memory deficits
Source: Mol Autism. 2020 Mar 30;11:22. doi: 10.1186/s13229-020-00328-3 (PMC7106595; doi:10.1186/s13229-020-00328-3)
Supplement: Supplementary file 1 — Additional file 1: Figure S1. The number of cortical neurons is unaltered in the adult Mir146a-/- brain. (a) Cortical slides of WT (top panel) and Mir146a-/- mouse (bottom panel) stained for NeuN to label post-mitotic neurons at P60. Number of NeuN+ cells normalized to area counted (±S.D.) in layer 2 (b), layers 3-4 (c), layer 6 (d) and all layers (e) in the WT and Mir146a-/- cortical slices. At least 2 images at comparable plane per mouse were analyzed. The number of NeuN+ cells at P30 and P60 are indistinguishable, thus, we combined the results for each genotype. Figure S2. Neurogenesis in the mouse neocortex. Neuroepithelial cells (NE) proliferate up to E11 before differentiating into apical radial glia (aRG). aRG are located in the ventricular zone (VZ) where their nucleus undergo interkinetic nuclear migration (INM) and divide at the apical surface. aRG either self-replicate or give rise to a neuron or an intermediate progenitor (IP) after an asymmetric division. IP remain in the subventricular zone (SVZ) and can complete one or two cell cycles before differentiating into neurons. Neurons coming from aRG or IP migrate towards the intermediate zone (IZ) and the cortical plate (CP) where they acquire their final position and identity and start expressing post-mitotic markers such as NeuN. Figure S3. Number of PH3+ cells at apical surface of the E14.5 neocortex is unaltered in the Mir146a-/-. Graph showing the number of PH3+ cells (±S.D.) in images shown in Fig. 1e. NS, not significant by Student’s 2-tailed T-Test. Figure S4. Loss of miR-146a does not affect cortical layers organization. (a) BrdU signal at P7 labeled neurons born at E14.5. The white box shows the analyzed area. (b) Zoom of the white box from (a). The 6 layers of the mature cortex were determined and BrdU+ cells were counted using 3 images from comparable plane. (c) Graph shows the percentage of cells in each layer for the two genotypes, normalized against the total number of labeled cells. Figure [file 13229_2020_328_MOESM1_ESM.pdf]

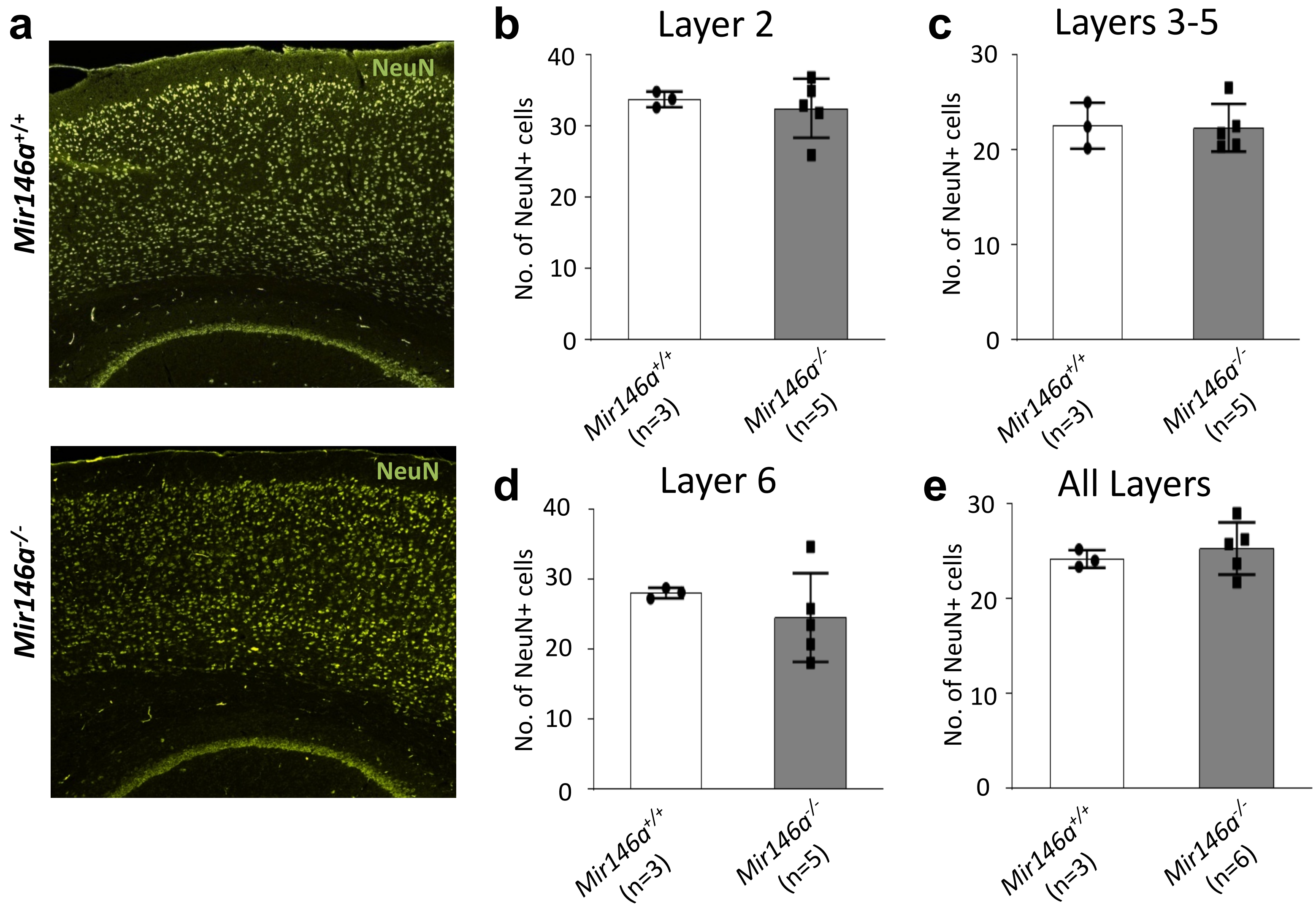

Figure S1, Fregeac *et al*

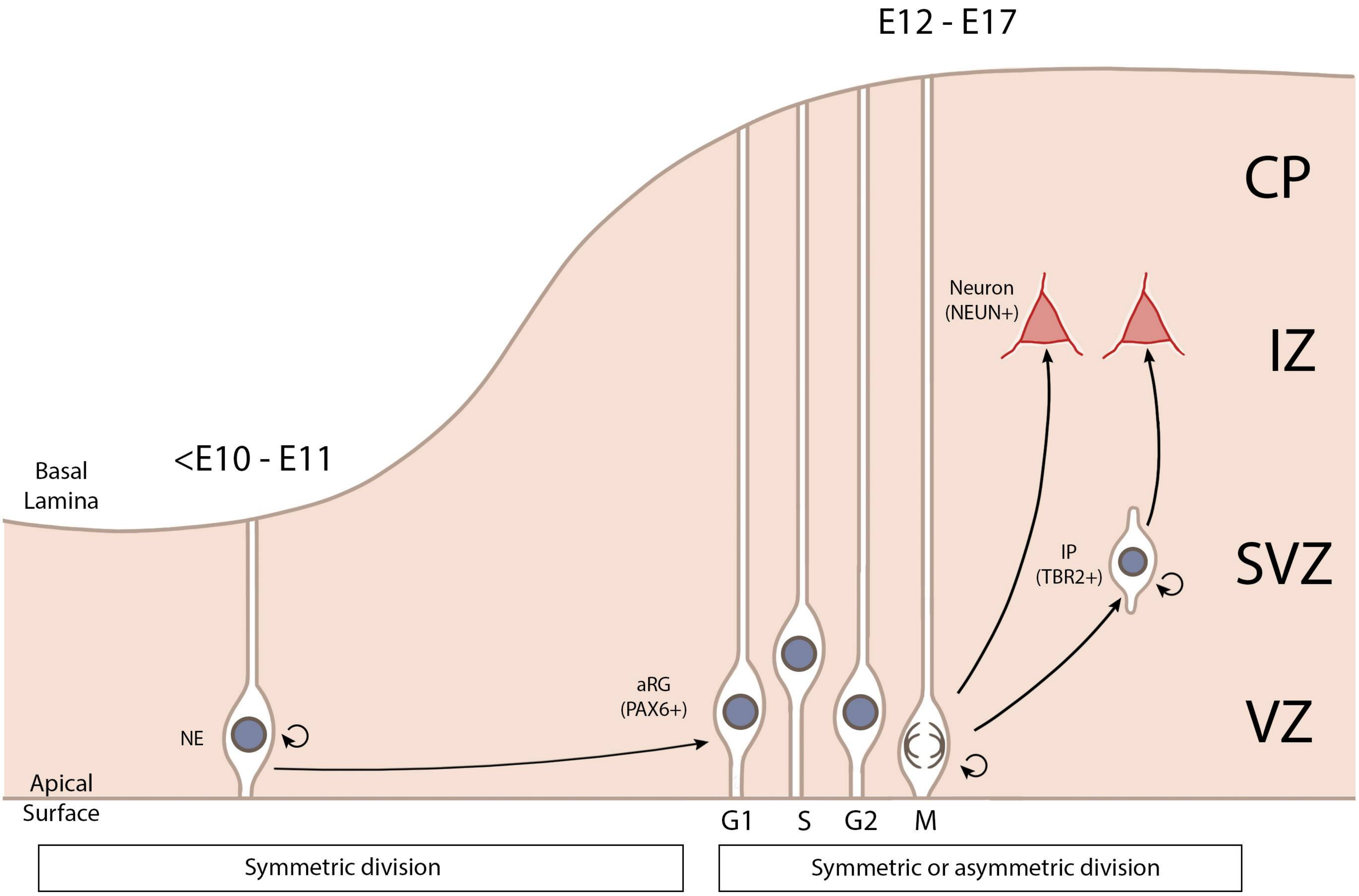

Figure S2, Fregeac *et al*

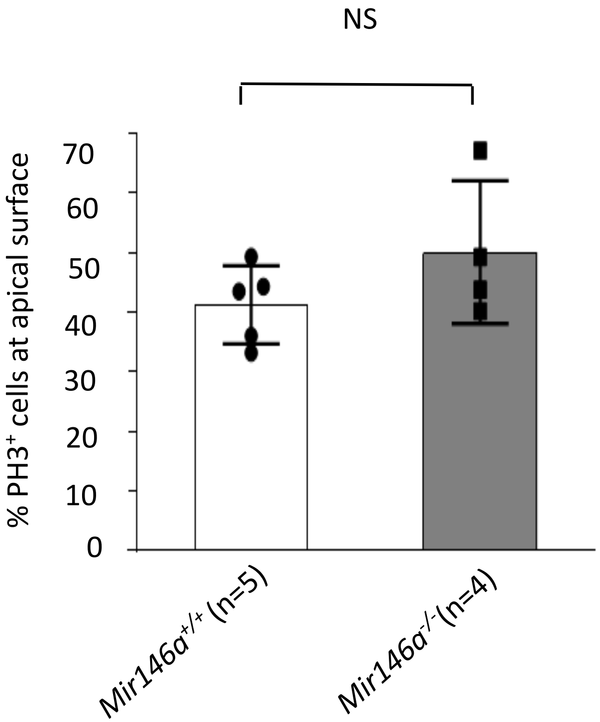

Figure S3, Fregeac *et al*

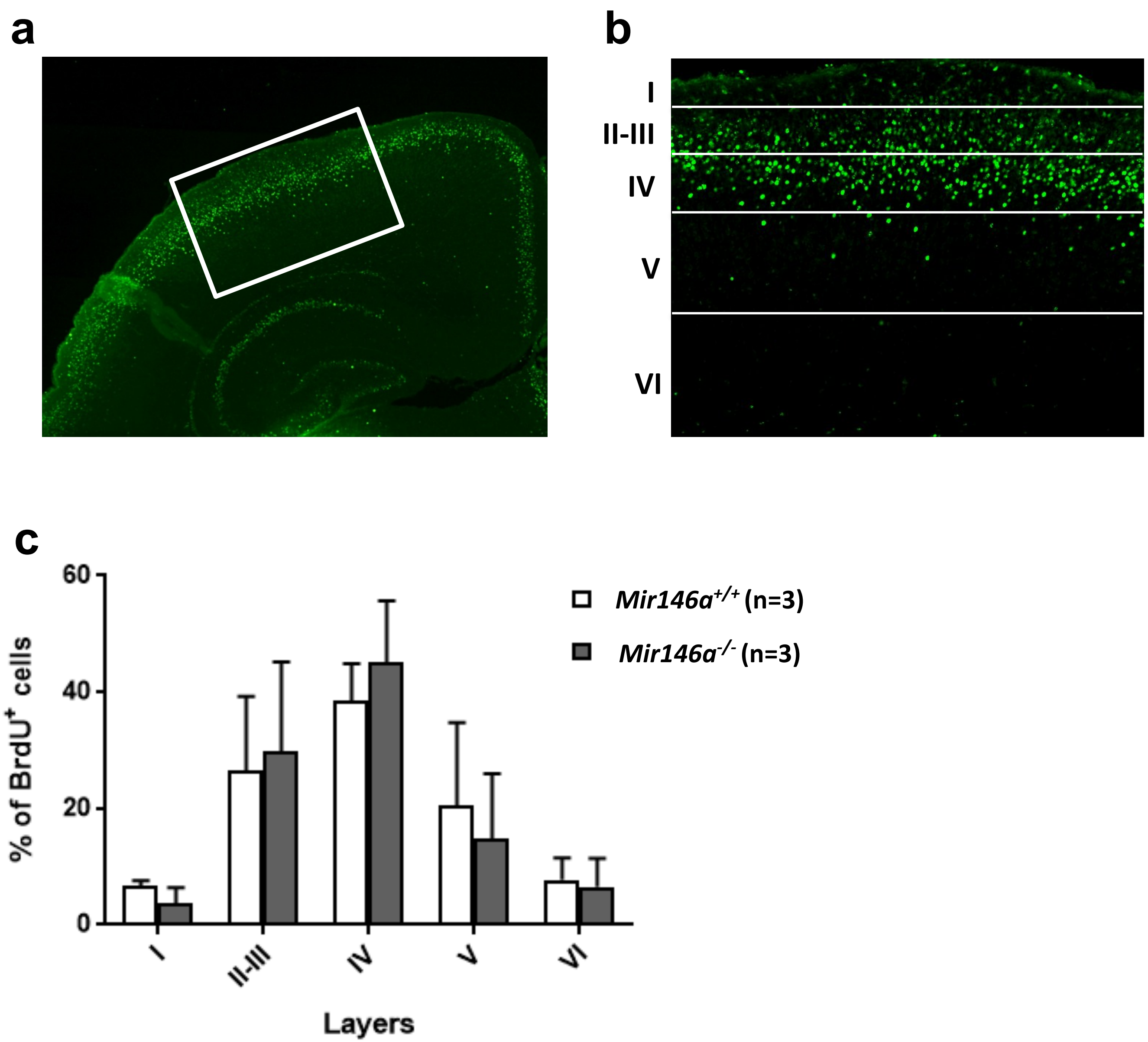

Figure S4, Fregeac *et al*

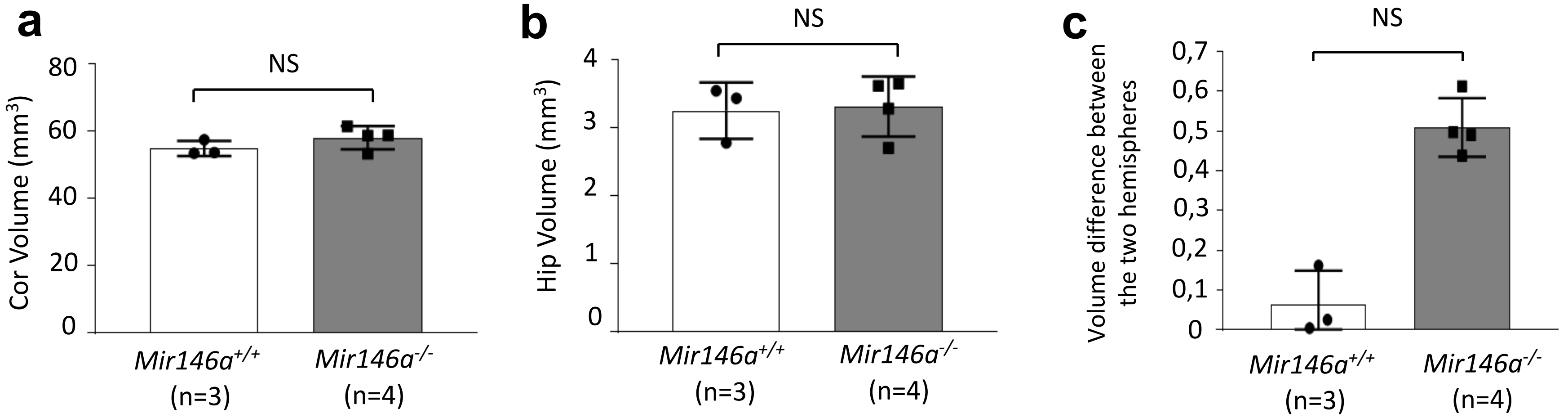

Figure S5, Fregeac *et al*

a

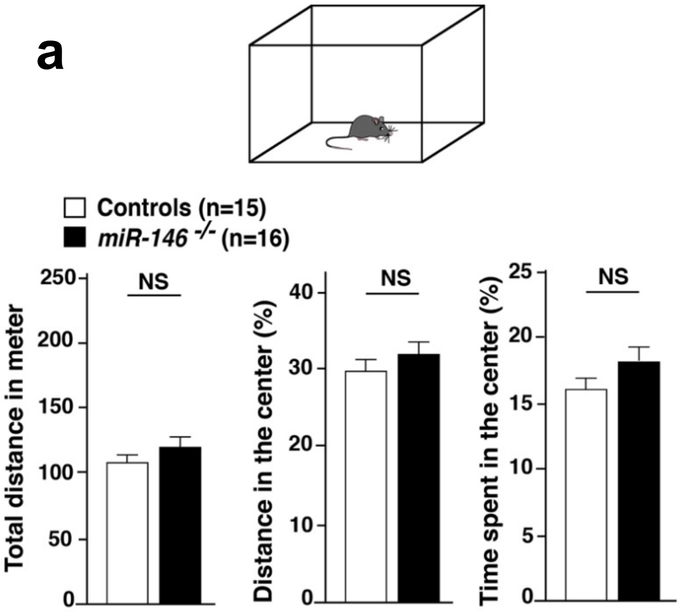

b

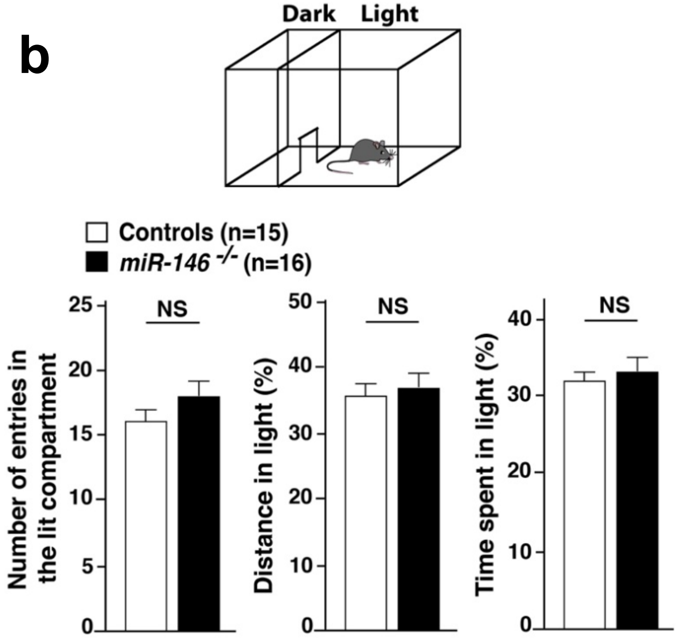

Figure S6, Fregeac *et al*

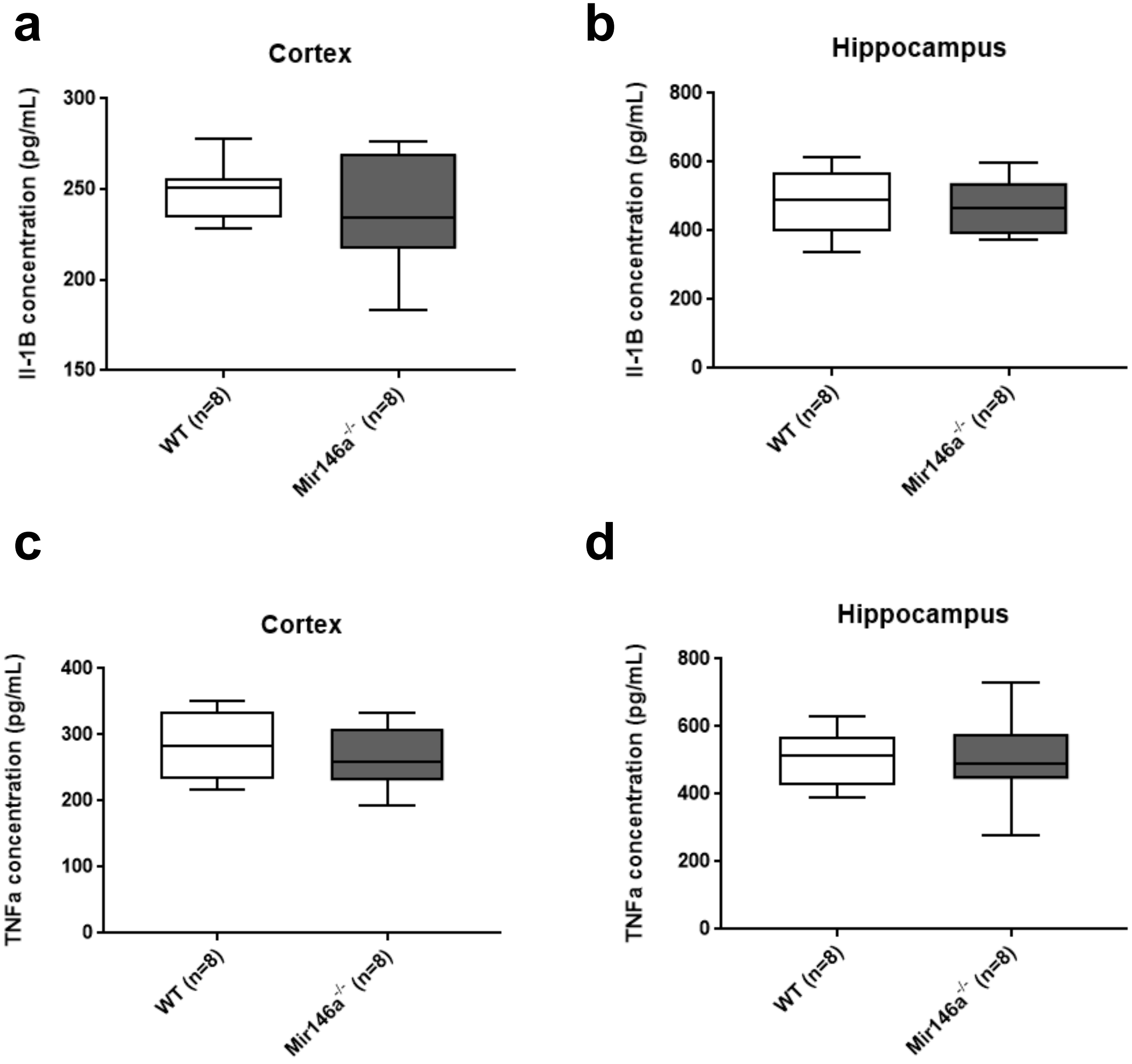

Figure S7, Fregeac *et al*
